# Recombinant Adeno-Associated Virus Vector Mediated Gene Editing in Proliferating and Polarized Cultures of Human Airway Epithelial Cells

**DOI:** 10.1089/hum.2024.260

**Published:** 2025-08-04

**Authors:** Soo Yeun Park, Zehua Feng, Soon H. Choi, Xiujuan Zhang, Yinghua Tang, Grace N. Gasser, Donovan Richart, Feng Yuan, Jianming Qiu, John F. Engelhardt, Ziying Yan

**Affiliations:** ^1^ Department of Anatomy and Cell Biology, Carver College of Medicine, University of Iowa, Iowa City, Iowa, USA; ^2^ Department of Microbiology, Molecular Genetics and Immunology, University of Kansas Medical Center, Kansas City, Kansas, USA.

**Keywords:** AAV, gene editing, airway basal cells, CFTR, reporter cell line

## Abstract

Cystic fibrosis (CF) is caused by mutations in the *cystic fibrosis transmembrane conductance regulator* (*CFTR*) gene. While CRISPR-based *CFTR* editing approaches have shown proof-of-concept for functional rescue in primary airway basal cells, induced pluripotent stem cells, and organoid cultures derived from patients with CF, their efficacy remains suboptimal. Here, we developed the CuFi^Cas9(Y66S)eGFP^ reporter system by integrating spCas9 and a non-fluorescent Y66S eGFP mutant into CuFi-8 cells, an immortalized human airway epithelial cell line derived from a patient with CF with homozygous F508del mutations. These cells retain the basal cell phenotype in proliferating cultures and can differentiate into polarized airway epithelium at an air-liquid interface (ALI), enabling both visualized detection of gene editing and electrophysiological assessment of *CFTR* functional restoration. Using this system, recombinant adeno-associated virus (rAAV)-mediated homology-directed repair (HDR) was evaluated in proliferating cultures. A correction rate of 13.5 ± 0.8% was achieved in a population where 82.3 ± 5.6% of cells were productively transduced by AAV.eGFP630g2-CMVmCh, an rAAV editing vector with an mCherry reporter. Dual-editing of F508del *CFTR* and Y66S *eGFP* was explored using AAV.HR-eGFP630-F508(g03) to deliver two templates and single guide RNAs. eGFP^+^ (Y66S-corrected) cells and eGFP^−^ (non-corrected) cells were sorted via fluorescence-activated cell sorting and differentiated at an ALI to assess the recovery of CFTR function. Despite a low F508 correction rate of 2.8%, ALI cultures derived from the eGFP^−^ population exhibited 25.2% of the CFTR-specific transepithelial Cl^−^ transport observed in CuFi-ALI cultures treated with CFTR modulators. Next-generation sequencing revealed frequent co-editing at both genomic loci, with sixfold higher F508 correction rate in the eGFP^+^ cells than eGFP^−^ cells. In both populations, non-homology end joining predominated over HDR. This reporter system provides a valuable platform for optimizing editing efficiencies in proliferating airway basal cells, particularly for development of strategies to enhance HDR through modulation of DNA repair pathways.

## INTRODUCTION

Cystic fibrosis (CF) is a recessive inherent life-threatening disease caused by mutations in the *cystic fibrosis transmembrane conductance regulator* (*CFTR*) gene.^1,2^ Dysfunctional or absent CFTR expression disrupts airway surface liquid (ASL) hydration, leading to thickened mucous layer that impairs mucociliary clearance and weakens innate immunity.^3^ In CF lungs, this creates an environment conducive to spontaneous bacterial colonization, ultimately resulting in chronic infection and progressive pulmonary failure, the primary cause of death in patients with CF.^4,5^ Gene therapy targeting CF lung disease is considered a promising path toward a cure,^6,7^ particularly as advances in the field have successfully treated certain genetic diseases.^8^ However, early CF clinical trials using recombinant adeno-associated virus (rAAV)^9,10^ and liposome-formulated plasmid^11^ as *CFTR* transfer agents have not yet achieved the desired outcomes. One primary factor attributed to these unsuccessful trials is the previous lack of efficient delivery vehicles for *CFTR* transfer to the airways.

Recent developments in human airway-tropic viral vectors have partly addressed this challenge. An ongoing CF clinical trial (NCT06526923) employs rAAV2.5T to deliver a shortened *CFTR* minigene with a 156-bp partial deletion at the regulatory (R) domain.^12^ rAAV2.5T is an AAV variant developed through directed evolution of an AAV capsid gene library in polarized human airway epithelium (HAE) cultured at an air–liquid interface (HAE-ALI).^13^ To address the package limitation of rAAV vectors, the chimeric parvoviral vector rAAV2/human bocavirus 1 (HBoV1) was developed. This vector is created by pseudopackaging the genome of rAAV2 into the capsid of HBoV1, a natural human respiratory pathogen. The large HBoV1 capsid can accommodate an oversized rAAV genome of up to 5.9 kb, enabling the delivery of a full-length *CFTR* coding sequence driven by a robust promoter.^14^

The advent of clustered regularly interspaced short palindromic repeats (CRISPR) genome editing has propelled gene therapy beyond traditional gene addition for functional complementation. Therapeutic gene editing approaches enable precise and permanent correction at the genome level to address the root cause of a genetic disease.^15^ Several CRISPR-based therapies to treat genetic diseases are currently in clinical trials.^16–18^ In CF, gene editing holds particular promise because correcting the defective *CFTR* at the genome level preserves its natural expression patterns, which is thought to be crucial for restoring CF lung function.^10^ If gene editing can target the airway progenitor cells that are capable of self-renewal and differentiation into various CFTR-expressing cell types, such as basal cells and club cells in the respiratory trees and alveolar type II cells in lung parenchyma,^19,20^ permanent and lasting gene correction may be achievable.^21^ Notably, rAAV2.5T has demonstrated its ability to transduce airway basal cells in HAE-ALI cultures *in vitro*.^22,23^ Inhale delivery of rAAV2.5T to the lungs of wild-type and CF ferrets demonstrated that pre-existing mucus in the CF ferrets did not present a barrier to effective transduction^24^ and preliminary data from our studies suggest that rAAV2.5T also can transduce the basal cells in ferret lungs *in vivo* (data not shown), highlighting its potential for testing *in vivo* CFTR gene editing in the CF ferret models.^25–27^

The CRISPR complex, consisting of a CRISPR-associated protein (Cas) and a single guide RNA (sgRNA), acts as a programmable endonuclease or nickase to induce double-stranded (ds) DNA breaks (DSB) or single-stranded (ss) DSB at predetermined sites. The subsequential repair of these chromosome lesions exploits cellular DNA repair pathways: non-homologous end joining (NHEJ) and homologous recombination (HR).^28^ Gene editing strategies leveraging these mechanisms to correct the mutations at the *CFTR* locus include homology-directed repair (HDR)^27,29–32^ and NHEJ-based homology-independent targeted insertion (HITI).^29^ Advanced editing tools of base editors and prime editing,^33–35^ as well as targeted insertion of a *CFTR* minigene expression cassette into a genome safe harbor or *CFTR* locus^36,37^ have also been explored for combating CF. These approaches have demonstrated proof-of-concept for functional rescue in primary airway basal cells, induced pluripotent stem cells, and organoid cultures derived from patients with CF. However, their overall efficacies remain limited. This underscores the need for a convenient, reproducible, and easily assessable reporter system to optimize and advance these techniques effectively.

CuFi-8 is an immortalized human CF airway epithelial cell line derived from a patient with CF with a homozygous F508del genotype.^38^ This cell line was established by introducing the expression of human telomerase reverse transcriptase and HPV-16 E6/E7 oncogenes. CuFi-8 cells maintain a basal cell phenotype in proliferating cultures and can differentiate into pseudostratified mucociliary epithelia when cultured at an ALI. Polarized CuFi-8 ALI cultures (CuFi-ALI) have been widely utilized to study *CFTR* gene transfer and its functional complementation in correcting CFTR-specific Cl^−^ transport deficiency.^14,39^ Furthermore, genetically modified CuFi-8 cells have been employed to study the airway transduction biology of rAAV2.5T^40^ and to explore host-virus interactions during HBoV1 infection *in vitro*.^41^ In this study, we established a CuFi-8-derived reporter cell line to evaluate rAAV-mediated gene editing in both proliferating and polarized airway epithelial cultures.

## MATERIALS AND METHODS

### Viral vectors and productions

#### Lentiviral vector transfer plasmid

pLenti-CMVCas9-p2a-Y66SeGFP-Puro was constructed using the elements from LentiCas9-Blast (Addgene plasmid #52962), which expresses a self-cleaving fusion protein of SpCas9 and BSD (product of the blasticidin S resistance gene)^42^ and pLenti CMV GFP Puro (658-5) (Addgene plasmid #17448).^43^ The *BSD* sequence in the cassette of SpCas9-p2A-BSD was replaced with the coding sequence for *Y66S eGFP*.^44^ The resultant sequence SpCas9-p2A-Y66SeGFP was cloned into pLenti CMV GFP Puro (658-5), replacing the sequence encoding wild-type *eGFP*.

#### rAAV vector transfer plasmids

pAAV2.eGFP630g2-CMVmCh is a derivative of pAAV2.tempG551DY66-gRNA(2),^27^ where the homologous template and sgRNA for HDR of the G551D mutation of ferret *CFTR* was replaced with the human cytomegalovirus immediate early promoter (CMV promoter)-driven mCherry reporter expression cassette. pAAV2.HR-eGFP630-F508(g0x) is another derivative of pAAV2.tempG551DY66-gRNA(2), where the elements for ferret G551D *CFTR* correction were replaced with an HDR template and an U6-promoter-sgRNA expression cassette (g0x = g01, or g01, or g03 sgRNA) enabling dual-correction of Y66S *eGFP* and F508del *CFTR*. Three sgRNA recognition sequences: g01, g02, and g03, positioned around or at the target site of *CFTR* exon 11 were chosen using the online tool CRISPOR (http://crispor.tefor.net). The recognition sequences of these sgRNAs are listed in Table 1. While these sgRNAs appeared active in the *in vitro* cleavage assay, only the g03 worked effectively in rAAV-transduced cells. The HDR template is a 995-bp sequence homologous to the F508del *CFTR* sequence in CuFi-8 cells encompassing the exon 11 but with a difference of 8 nucleotides, including the insertion of three nucleotides (CTT) at the I507 codon (AT//T) to rebuild the codon for F508 (AAC-TTT, I507-F508) and five additional nucleotides silent substitutions. Specifically, one base was altered 35-bp upstream of the target site for g01, while three bases were changed 7-bp downstream of the target site for g03. These substitutions disrupt the g01 and g03 sgRNA recognition sequences in the HDR template and prevent subsequent CRISPR cleavage at the HDR-edited sequences. Additionally, a *Kpn*I restriction site, absent in the original allele, was introduced 20-bp upstream of the target site to facilitate genotype verification. Notably, g02 does not recognize the HDR template and the corrected sequence, where the inclusion of the F508 codon disrupts its protospacer adjacent motif (PAM).

**Table 1.** Sequence of Mutagenesis Template, Single Guide RNA Targeted Site, and PCR Primers

Category	Name	Sequence
1,060 kb *CFTR*	CuFi-Fw	TGGAGGCAAGTGAATCCTGAGC
	CuFi-Rev	GGGTAGTGTGAAGGGTTCATATGC
308 bp amplicon	e11Fw	CACATAGAACAGCACTCGACACAGAGT
	e11Rev	TGGTATTTGTTCAAAGCCAGGGAT
763 bp *eGFP*	Pfw	AGAATCCTGGACCGATGGTG
	Prev	TCCAGAGGTTGATTGTCGACG
sgRNA (Y66S *eGFP*)	g2	gcggctgaagcactgca//cgcCGG
sgRNA (F508del *CFTR*)	g01	aatggtgccaggcataa//tccAGG
	g02	accattaaagaaaatat//catTGG
	g03	tctgtatctatattcat//catAGG
Primer/probe set (*Cas9*)	Cas9-Fw	CCCAAGAGGAACAGCGATAAG
	Cas9-Rev	CCACCACCAGCACAGAATAG-
	Cas9-Pro	atcgccagaaagaaggactgggac
Primer/probe set (*eGFP*)	GFP-Fw	GTGAACCGCATCGAGCTGAA
	GFP-Rev	TGCTTGTCGGCCATGATATAG
	GFP-Pro	atcgacttcaaggaggacggcaac
Primer/probe set (*GAPDH*)	GAP-Fw	CTTTGGTATCGTGGAAGGACTC
	GAP-Rev	GTAGAGGCAGGGATGATGTTC
	GAP-Pro	cgggaaactgtggcgtgatg
Primer/probe set (U6 pro)	U6-Fw	AGGGCCTATTTCCCATGATTC
	U6-Rev	AAACTGCAAACTACCCAAGAAA
	U6-Pro	ttgcatatacgatacaaggctgttagagaga

In single guide RNA sequences, upper cases are the protospacer adjacent motif, // mark the cleavage site.

The TaqMan probes are labeled with 6-carboxyfluorescein (FAM) at the 5′-end as the reporter, and tagged with Dark Hole Quencher 1 (BHQ1) at the 3′-end as the quencher.

CFTR, cystic fibrosis transmembrane conductance regulator; sgRNA, single guide RNA.

#### Vector production

VSV-G pseudotyped lentiviral vectors were generated from transfection in HEK293T cells. Functional titers as transducing units (TU)/mL were quantified using TaqMan PCR quantification of viral genome integration following infection of HEK293T, as previously described.^45^

rAAV6 and rAAV2.5T vectors were generated from triple transfection of the rAAV2 proviral transfer plasmid with two helper plasmids pAd4.1 and pAAVRep2Cap6 or pAAVRep2Cap2.5T, as previously described.^46^ rAAV2/HBoV1.eGFP630g2-CMVmCh and rAAV2/HBoV1.CBACFTR^14^ were generated by pseudopackaging a rAAV2 genome into HBoV1 capsid using the HBoV1-NS-free package system.^47^ The titers of these vectors were determined by TaqMan PCR as DNase I-resistant particles (DRP)/µL.

### Cell cultures

#### Proliferating cultures

CuFi-8 cells were cultured and passaged in PneumaCult^TM^-Ex Plus medium (StemCell Technologies, Vancouver, Canada) on plastic plates or dishes precoated with collagen IV (Sigma, St. Louis, MO).

#### Polarized CuFi-8 epithelial cultures

In total, 1.5 × 10^5^ CuFi-8 cells were seeded onto 6.5-mm, polyester Transwell® inserts (#3470, Corning, Corning, USA) that were precoated with collagen IV. Seeding occurred in PneumaCult^TM^-Ex Plus medium. At 24 h post-seeding, the medium was replaced with PneumaCult^TM^ ALI medium (StemCell Technologies) in both apical and basal chambers. Cultures were then air-lifted the following day to facilitate differentiation at ALI for 3 weeks.^48^ The basal chamber medium was replenished every other day in the first week then twice a week during the culturing period. Matured polarized airway epithelium cultures (CuFi-ALI) with a transepithelial electrical resistance (TEER) >1,000 Ω/cm^2^ were used for Ussing chamber assays.^49^ In polarized HAE cultures, rAAV apical transduction encounters post-entry barriers that limit nuclear transport.^50,51^ Doxorubicin (Dox) was utilized to enhance the apical transduction of rAAV6 and rAAV2/HBoV1 in ALI cultures derived from CuFi-8 cells and their derivative reporter cells. Dox (2.5 µM) was added to the basal chamber medium during the transduction period (16 h). Its presence facilitated rAAV nuclear transport, promoting productive transduction in polarized HAE-ALI.^52^

#### Generation of the CuFi^Cas9(Y66S)eGFP^ cells

Proliferating CuFi-8 cells were seeded onto a well of six-well plate at the density of 2 × 10^5^ cells per well. The day after seeding, cells were transduced with Lent-CMVCas9-p2a-Y66SeGFP-Puro at a multiplicity of infection (MOI) of 1.5 TU/cell. Selection for puromycin (Puro) resistant cells was started 2 days after lentiviral infection in PneumaCult^TM^-Ex Plus medium supplemented with 1 µg/mL Puro. All cells of mock-infected control died within 2 days after exposure to Puro, allowing for rapid selection of a Puro-resistant polyclonal pool within a week, expressing spCas9-p2a and Y66SeGFP proteins. The conditions for proliferating and polarized ALI cultures of CuFi^Cas9(Y66S)eGFP^ cells were the same as for CuFi-8 cells.

### Gene editing

CuFi^Cas9(Y66S)eGFP^ cells were seeded onto a six-well plate at a density of 2 × 10^5^ cells/well. The next day, the cells were transduced with AAV2/6.eGFP630g2-CMVmCh at various MOIs. Three days later, transduction efficiency and Y66S eGFP correction efficiency were determined by fluorescence-activated cell sorting (FACS) at the Flow Cytometry Facility of the University of Iowa, using the BD® LSR II Flow Cytometer (Franklin Lakes, NJ). The ALI cultures derived from CuFi^Cas9(Y66S)eGFP^ cells were apically transduced with AAV2/HBoV1.eGFP630g2-CMVmCh at MOI of at an MOI of 5 × 10^4^ DRP/cell (MOI of 50K). In total, 2.5 µM Dox was added in the culture medium of the basal chamber during the 16 h infection period. At 7 days post-transduction, cells were lysed for genomic DNA extraction, PCR, Topo-cloning, and Sanger sequencing were used to assess gene editing at the Y66S *eGFP* locus.

To assess the dual-correction of the Y66S *eGFP* and F508del *CFTR*, CuFi^Cas9(Y66S)eGFP^ cells were transduced with AAV2/2.5T.HR-eGFP630-F508 at an MOI of 100K. Two days post-transduction, a portion of the transduced cells was used for flow cytometry to determine the efficiency of Y66S eGFP correction and lysed for extraction of genome DNA. The remaining rAAV-transduced cells were either transferred to Transwell® inserts for ALI cultures or expanded on a 100-mm dish. After 5 days, the expanded cultures were subjected to FACS at the Flow Cytometry Facility of University of Iowa, using the Cytek Aurora^TM^ CS system (Fremont, CA). Viable Y66S *eGFP*-corrected cells (green) and a population of non-green cells were collected and further expanded for a week to obtain enough cells used for polarized ALI cultures. A subset of cells from each cell population was lysed for genomic DNA extraction.

### Amplicon-EZ sequencing

Genomic DNA extracted from rAAV transduced cells (the unsorted cells at 2 days post-infection or the sorted eGFP^+^ and eGFP^−^ cells) was used for nested PCR to obtain the library for amplicon sequencing. A PCR primer set CuFi-Fw/CuFi-Rev, located outside the *CFTR* homologous sequence carried in AAV2.HR-eGFP630-F508, was used to amplify a 1.06-kb DNA product spanning the target site, excluding the rAAV viral DNA. Next, a 308-bp amplicon was generated by using the primer set e11Fw/e11Rev (with Illumina adapters) and the 1.06-kb PCR product as a template. Amplicons were evaluated by next-generation sequencing (NGS) to quantify variants using an Illumina platform. Amplicon-EZ sequencing and analyses were conducted at Azenta Life Sciences USA Inc. (South Plainfield, NJ).

### Short-circuit current measurements in Ussing chambers

The ALI culture inserts were placed under VCC MC8 voltage/current clamps in self-contained P2300 Ussing chambers (Physiologic Instruments, San Diego, CA, USA) for short-circuit current (*I*sc) measurement using an asymmetrical chloride buffer system, as previously described.^14^ In total, 100 μM amiloride, 100 μM 4,4’-diisothiocyano-2,2’-stilbenedisulfonic acid (DIDS), 100 μM 3-isobutyl-1-methylxanthine (IBMX)/10 μM forskolin (Forsk), and 50 μM GlyH-101 were sequentially added to the apical chamber, and the changes in current were recorded during the experiment.

### Primers for genotyping and quantitative PCR analysis

Sequences of PCR primers and the TaqMan primer/probe sets for *eGFP*, *CFTR*, *Cas9*, and *GAPDH* are listed in Table 1. TaqMan probe-based quantitative PCR was used to quantify the titers of the viral vector stocks and the integrated lentiviral genome copies (*Cas9* and *eGFP*), normalized to cellular genes *CFTR* and *GAPDH*. The TaqMan probes were tagged with 6-carboxy fluorescein (FAM) at the 5′-end as the reporter and with Dark Hole Quencher 1 (BHQ1) at the 3′-end as the quencher. The quantitative PCR reactions were performed and analyzed using the Bio-Rad My IQTM Real-time PCR detection system and software (Bio-Rad, Hercules, CA). Standard curves were generated using known amounts of plasmids harboring the corresponding DNA fragments to calculate copy numbers.

### Immunofluorescence analysis and antibodies for epithelial cell type markers

For whole mount staining, Transwell® inserts were washed with phosphate-buffered saline (PBS) and fixed with 4% paraformaldehyde (ThermoFisher Scientific, Waltham, MA) for 30 min, followed by three washes with PBS. Immunostaining of the epithelial cell layer on the supportive membrane was performed within the inserts. For Cryosection (10 µm) slides, supportive membranes cut from the Transwells were fixed, washed with PBS, and embedded in the Tissue-Plus® O.C.T. Compound (23-730-571, Fisher HealthCare, Houston, TX). Immunostaining of cells on the membrane of the inserts and cryosection slides followed the same procedure, as described below. To permeabilize the cell membrane, PBS containing 2% Triton X-100 was applied for 30 min at room temperature, followed by three washes with PBS. Samples were then blocked with the blocking solution (20% of Donkey serum, 0.1% Triton X-100, 0.1 mM in CaCl_2_ in PBS) for 1 h at room temperature. Primary antibodies diluted in donkey diluent (1% donkey serum, 0.1% Triton X-100, 0.1 mM CaCl_2_ in PBS) were applied overnight at 4°C. The following day, after three washes with PBS, secondary antibodies diluted in donkey diluent were applied and incubated for 1 h at room temperature. Hoechst 33342 (ThermoFisher Scientific) was added for nuclei staining. After washing with PBS, the supportive membranes were cut from the Transwells using a scalpel and mounted onto the slides with the apical side facing up. ProLong^TM^ Gold Antifade Mount (Invitrogen^TM^, ThermoFisher Scientific) was applied onto the membrane, as well as the sample of the cryosection slides, which was then covered with a coverslip. Slides were examined under a Zeiss LSM 880 confocal microscope.

The primary antibodies for cell markers were included:^53,54^ anti-acetylated tubulin (1:500; T7451, Sigma-Aldrich, St. Louis, MO), anti-BSND (1:500; ab196017, Abcam, Waltham, MA), anti-TP63 (1:175; AF1916, R&D Systems, Minneapolis, MN), anti-Keratin 5 (1:500; 905501, Biolegend, San Diego, CA), anti-Mucin 5AC (1:500; ab3649, Abcam, Waltham, MA), and anti-Uteroglobin (1:4,000; ab213203, Abcam, Waltham, MA). The secondary antibodies included: Alexa Fluor® 488-conjugated AffiniPure® Donkey Anti-Chicken IgY (IgG) (H + L) (1:250; 703-546-155, Jackson ImmunoResearch, West Grove, PA), Alexa Flour® 488-conjugated AffiniPure® donkey anti-rabbit IgG (1:250; A21206, Invitrogen^TM^, ThermoFisher Scientific), Alexa Fluor® 488-conjugated AffiniPure® donkey anti-mouse IgG (1:250; A21202, Invitrogen^TM^, ThermoFisher Scientific), Alexa Fluor 568-conjugated AffiniPure® donkey anti-goat IgG (1:250; A-11057, Jackson ImmunoResearch, West Grove, PA), Alexa Fluor® 555-conjugated AffiniPure® Donkey Anti-Rabbit IgG (H + L) (1:250; A31572, Life Technologies, Invitrogen^TM^, ThermoFisher Scientific), Alexa Fluor® 647-conjugated AffiniPure® F(ab’)_2_ Fragment Donkey Anti-Chicken IgY (IgG) (H + L) (1:250; 703-606-155, Jackson ImmunoResearch, West Grove, PA), and Alexa Fluor® 647-conjugated AffiniPure® F(ab’)_2_ Fragment Donkey Anti-Rabbit IgG (H + L) (1:250; 715-606-151, Jackson ImmunoResearch).

### Statistical analysis

Statistical analysis was performed by using GraphPad Prism 8. Values and error bars show means ± standard deviation, statistical significance was determined using a Student’s *t-*test or analysis of variance, or Pearson correlation with *p* < 0.05 being significant.

## RESULTS

### Genetically modified CuFi-8 cells maintain the potential to be differentiated into polarized airway epithelia

**Figure 1.**
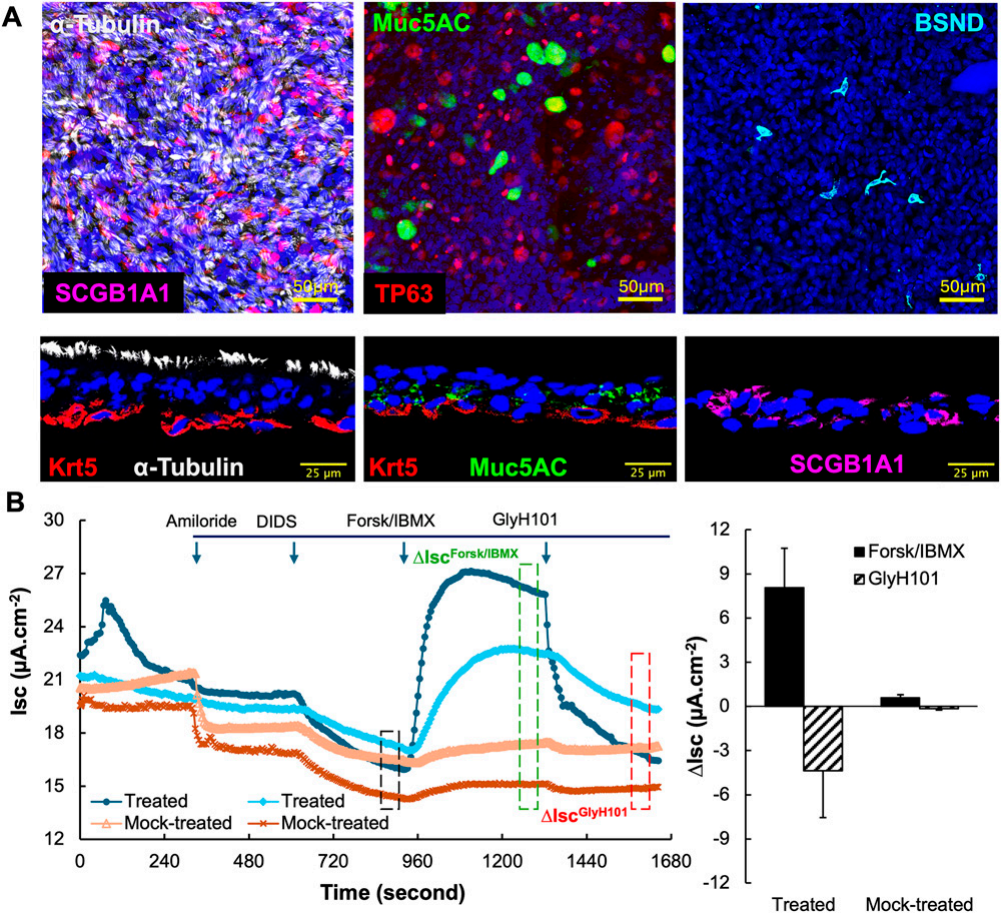
Polarized airway epithelial cultures form CuFi-8 cells at an ALI. **(A)** Major cell types. CuFi-ALI cultures were stained with antibodies against the marker of major epithelial cell type: α-tubulin (ciliated cells), Muc5AC (goblet cells), SCGB1A1 (Club cells), TP63 or Krt5 (basal cells), and BSND (ionocytes). Hoechst 33342 was used to visualize nuclei (*blue*). En-face images were captured at the center of the transwells at 20×, using a Zeiss 770 confocal microscope. The polar composition of the typical epithelial cell types is shown with the representative images of the stained cross-section of the culture in a supported membrane embedded in OCT compound. **(B)** Functional characterization of F508del CFTR expression in polarized CuFi-8 ALI cultures. CFTR channel activity was assessed in polarized CuFi-ALI cultures using Ussing chamber measurements of short-circuit currents (*Isc*) to evaluate transepithelial Cl^–^ transport. Representative current traces recorded during the experiment are presented. After blocking epithelial sodium channels and chloride channels with amiloride and DIDS, respectively, CFTR activity was stimulated with the cAMP agonists IBMX/forskolin and inhibited with the CFTR inhibitor GlyH101. Changes in CFTR-dependent currents (Δ*Isc*^Forsk/IBMX^ and Δ*Isc*^GlyH101^) were quantified: Δ*Isc*^Forsk/IBMX^ as the mean plateaued current over 45 s post-agonist addition (*green box*) minus the mean baseline current before stimulation (*black box*), and Δ*Isc*^GlyH101^ as the mean plateaued current post-GlyH101 addition (*red box*) minus the agonist-induced current (*green box*). CFTR-specific transepithelial Cl^–^ transport was detected in the ALI cultures treated with the CFTR modulators VX770/VX809, whereas only background levels were recorded in the vehicle (DMSO) mock-treated controls. Data are presented as mean ± SD, with *N* = 3 independent cultures for the treated group and *N* = 4 for the mock-treated group. ALI, air–liquid interface; CFTR, cystic fibrosis transmembrane conductance regulator; DIDS, 4,4’-diisothiocyano-2,2’-stilbenedisulfonic acid; SD, standard deviation.

When cultured at an ALI, CuFi-8 cells can differentiate into multiple cell types that compose the proximal airway epithelium and regulate ASL fluid dynamics. These cells include ciliated cells, goblet cells, club cells, basal cells, and pulmonary ionocytes (despite being rare in the population), as shown by staining for cell markers of α-tubulin (ciliated cells), Muc5AC (goblet cells), SCGB1A1 (club cells), Krt5 or TP63 (basal cells), and BSND (ionocytes) (Fig. 1A). Additionally, the integrity of the mutated *CFTR* exon 11 in the homozygous F508del genotype was confirmed by PCR followed by Sanger sequencing. To verify the expression of the dysfunctional F508del CFTR protein in polarized CuFi-ALI cultures, we employed an epithelial voltage clamp and a self-contained Ussing chamber system to monitor changes in transepithelial short-circuit current (*I*sc) following sequential additions of ion channel blockers or agonists as previously described.^39^ In this electrophysiologic assay, amiloride was first applied to block ENaC channels, followed by DIDS to inhibit non-CFTR anion channels. CFTR channel activity was then assessed by the increase in Isc (Δ*I*sc^Frosk/IBMX^) induced by cAMP agonists IBMX and forskolin, which was subsequently inhibited by CFTR-specific blocker GlyH101. The CFTR-specific transepithelial Cl^−^ transport was quantified as Δ*I*sc^GlyH101^. In this experiment, prior to *Isc* measurement in Ussing chamber, CuFi-ALI cultures were treated with CFTR modulators VX770 and VX809 or mock-treated with vehicle Dimethylsulfoxide (DMSO).^55^ As expected, mock-treated CuFi-ALI cultures showed undetectable levels of Δ*I*sc^GlyH101^. In contrast, treatment with CFTR modulators led to detectable CFTR-specific Cl^−^ transport (Fig. 1B), indicating that polarized CuFi-ALI cultures produced F508del CFTR protein, which fails to traffic to the plasma membrane without the intervention of CFTR modulators. The CFTR modulators VX-770 and VX-809 synergistically rescue the defective assembly of F508del CFTR’s cytoplasmic nucleotide-binding domains and overcome its intracellular trafficking and gating defects, enabling functional expression on the apical membrane.^56^ Therefore, we confirmed that the CuFi-ALI cultures expressed the dysfunctional F508del CFTR protein and concluded that the CuFi-8 cell line is suitable for *CFTR* gene editing research, as correcting the F508del mutation at genome level would restore the CFTR-specific transepithelial Cl^−^ transport function through the production of normal CFTR protein.

**Figure 2.**
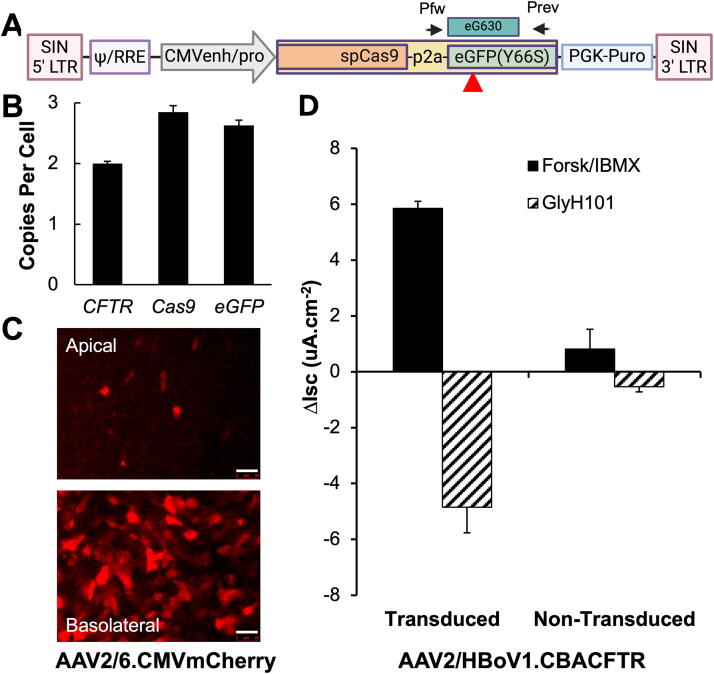
Genetically modified CuFi derivative cells retain differentiation potential when cultured at an air–liquid interface. **(A)** Schematic of the lentiviral vector. Lenti-CMVCas9-p2a-Y66SeGFP-Puro vector was used to transduce the proliferating CuFi-8 cell cultures for stable integration and expression of the self-cleaving fusion protein Cas9-p2a-Y66SeGFP. The Y66S eGFP is a non-fluorescent mutant protein caused by the Y66S mutation (indicated by the red triangle). The *cyan box* represents the sequence in the gene editing vectors for HR. The *black arrows* indicate the primer set for PCR amplification to assess mutation correction. **(B)** Integration of lentiviral genome copies. CuFi^Cas9(Y66S)eGFP^ reporter cells were obtained by lentiviral infection followed by puromycin selection. TaqMan quantitative PCR was used to determine the integrated viral genome copies compared with that of *CFTR* per cell. **(C)** Polar transduction of rAAV6 in ALI cultures polarized from CuFi^Cas9(Y66S)eGFP^ cells. ALI cultures were transduced with AAV2/6.CMVmCherry at the same MOI of 20K from the apical and basolateral membranes, respectively. Images were captured at 5 days post-infection. Scale bar: 50 µm. **(D)** Transduction of rAAV2/HBoV1 vector. The ALI cultures were apically transduced with rAAV2/HBoV1.CBACFTR at an MOI of 20K. Short-circuit currents (*Isc*) were measured in Ussing chamber 6 days post-infection. *Isc* of the CFTR-specific Cl^–^ transmembrane transportation was observed in the vector-transduced cultures. Data are presented as the mean ± SD, with *N* = 4 independent cultures in each condition. For the apical transductions of **(C)** and **(D)**, 2.5 µM doxorubicin was included in the basal chamber medium during the transduction period. HBoV1, human bocavirus 1; HR, homologous recombination; MOI, multiplicity of infection; rAAV, recombinant adeno-associated virus.

As CFTR expression is inefficient in airway basal cells and there is no convenient method for functional correction, an easily assessable reporter system is critical for optimizing gene editing approaches. To address this, we integrated stable expressions of Cas9 and a Y66S eGFP reporter into CuFi-8 cells using the lentiviral vector Lenti-CMVCas9-p2a-Y66SeGFP-Puro (Fig. 2A). Y66S eGFP is a non-fluorescent GFP mutant resulting from a single nucleotide (NT) substitution at the Y66 codon [TAC (tyrosine)→TCC (serine)] in the *eGFP* cDNA.^44^ Following puromycin (Puro) treatment, a Puro-resistant cell pool was obtained and designated as CuFi^Cas9(Y66S)eGFP^.

TaqMan probe-based quantitative PCR analysis of *Cas9* and *eGFP* genomic copies of the CuFi^Cas9(Y66S)eGFP^ cells revealed an average of 2.4 viral genome integrations per cell, normalized to the diploid genome with two copies of *CFTR* (Fig. 2B). CuFi^Cas9(Y66S)eGFP^ cells were then seeded onto Transwell® inserts and cultured at an ALI. After 3 weeks, the cultures exhibited a TEER exceeding 1,000 Ω/cm^2^, indicating the establishment of tight junctions and maturation of a well-differentiated epithelial layer. The polarization of the ALI cultures was also verified by the polarized rAAV6 transduction. rAAV6 vectors have demonstrated differential tropisms of transducing primary HAE-ALI cultures from the apical and basolateral membranes.^46^ At an MOI of 20K, AAV2/6.CMVmCherry efficiently transduced CuFi^Cas9(Y66S)eGFP^ ALI cultures from the basolateral membrane but inefficiently from the apical membrane (Fig. 2C). Further validation was conducted using apical transduction of AAV2/HBoV1.CBACFTR at an MOI of 20 K^14^; this vector contains an rAAV2 genome with an HBoV1 capsid that is highly tropic for the apical membrane of human airway epithelia. One-week post-transduction, CFTR-dependent Cl^−^ transport was measured. rAAV2/HBoV1 vector delivery partially restored CFTR-specific Cl^−^ transport deficiency in ALI cultures derived from CuFi^Cas9(Y66S)eGFP^ cells (Fig. 2D), with Δ*Isc*^GlyH101^ comparable with those observed in CFTR modulator-treated CuFi-8 ALI cultures. This result aligns with our previous report on the transduction efficiency of primary CF ALI cultures with rAAV/HBoV1.^14^ Notably, rAAV/HBoV1 selectively transduces HAE-ALI from the apical membrane but inefficiently transduces undifferentiated CuFi-8 cells,^14^ consistent with previous observations of HBoV1 infection efficiency in proliferating airway cells and polarized airway epithelium.^57^

Collectively, we introduced stable *Cas9* and Y66S *eGFP* integration into CuFi-8 cells via lentiviral transduction. This genetically modified CuFi-8 cell line (CuFi^Cas9(Y66S)eGFP^) maintains a multipotent basal cell-like ability to differentiate into major epithelial cell types in the proximal airway epithelium.

### Gene editing in CuFi^Cas9(Y66S)GFP^ cells

**Figure 3.**
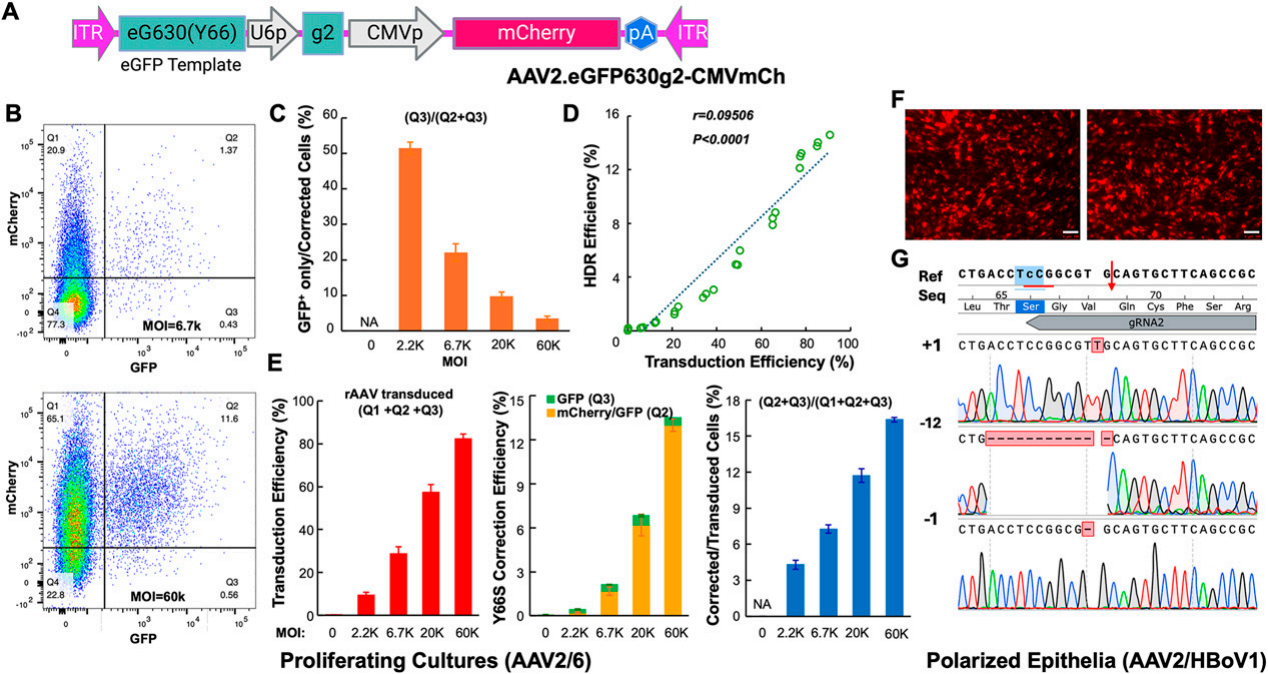
Homology-directed repair (HDR) of the Y66S mutation in CuFi^Cas9(Y66S)eGFP^ reporter cells. **(A)** Schematic of the rAAV construct used to correct the Y66S *eGFP*. sgRNA g2 recognizes the Y66S *eGFP* sequence, but it does not guide the CRISPR complex to cut the wild-type *eGFP* sequence and the template for HDR. The PAM of g2 overlapped with the Y66S codon. **(B–E)** Analyses of the rAAV-mediated HDR in proliferating CuFi^Cas9(Y66S)eGFP^ cells. Cells were transduced with the AAV2/6.eGFP630g2-CMVmCh at different MOIs. Flow cytometry was used to quantify the efficiencies of transduction and Y66S correction 5 days post-infection. **(B)** Flow cytometry separated the rAAV transduced cells into four populations. Q1: rAAV transduced but not succeed in Y66S mutation correction; Q2: Y66S mutation corrected with mCherry expression; Q3: Y66S mutation corrected without mCherry expression. Q4: not productively transduced by rAAV6. Representative analyses of two transductions with 6.7K and 60K MOIs, respectively, are shown. **(C)** Ratio of the cells expressing eGFP only (Q2) in all the Y66S corrected cells (Q2 + Q3). **(D)**. Scatter plot shows a significant positive correlation between the rAAV transduction efficiency [(Q1 + Q2 + Q3)/total counts] and the HDR efficiency [(Q2 + Q3)/total counts], *r* = 0.97055, *p* < 0.0001. Each dot represents a variable from FACS analysis, *N* = 31 (six independent transductions with four different MOIs [total 24] + seven non-transduced controls). **(E)** Plots show the dose-dependent rAAV transduction efficiencies and the Y66S correction efficiencies in the whole cell culture as well as in the rAAV transduced cell population. Data represent the mean ± SD of six (*N* = 6) independent transductions with indicated MOIs. **(F-G)** Gene editing in polarized ALI cultures differentiated from CuFi^Cas9(Y66S)eGFP^ cells. ALI cultures were apically transduced with AAV2/HBoV1.eGFP630g2-CMVmCh at an MOI of 50K. **(F)** mCherry reporter expression was visualized but eGFP expression (Y66S correction) was not detectable. Representative images captured 7 days post-transduction are shown. Scale bar: 100 µm. **(G)** Mutations resulted from NHEJ of DSBs at and around the CRISPR cut site were detected by PCR. PCR products across the Y66S codon from the AAV2/HBoV1-transduced ALI cultures were cloned into cloning vector pCR4Blunt-Topo and Sanger sequenced. Representative sequencing chromatograms show the products of (+1), (−12), and (−1) indels, aligned to the part of the reference sequence of Y66S *eGFP*. Gray box shows the g2 recognized sequence, *red arrow* points to the CRISPR cut site, and light blue box highlights the PAM sequence. HDR, homology-directed repair; NHEJ, non-homology end joining; PAM, protospacer adjacent motif; sgRNA, single guide RNA.

We constructed an rAAV vector, AAV2.eGFP630g2-CMVmCh (Fig. 3A), which harbors an HDR template encoding a 630-bp truncated *eGFP* sequence with the normal Y66 codon^58^ alongside an mCherry reporter cassette to index transduction efficiency. The vector also expresses an sgRNA, designated as g2 under the U6 promoter. This guide RNA (g2) specifically recognizes the mutant *eGFP* sequence where its PAM sequence overlaps with the Y66S codon; thus, the wild-type and gene-corrected Y66 sequences are not cleaved by Cas9/g2.^27^

Proliferating cultures of CuFi^Cas9(Y66S)eGFP^ cells were transduced with AAV2/6 eGFP630g2-CMVmCh at an MOI ranging from 2.2K to 60K at approximately threefold increments. Three days post-transduction, FACS was performed to quantify transduction (*i.e.,* mCherry expression) and Y66S correction efficiencies. Two representative flow cytograms from transductions with a ∼10-fold difference in MOIs are shown in Figure 3B. FACS identified four populations: Q4 (mCherry^−^/eGFP^−^, the cells not productively transduced by rAAV6), Q1 (mCherry^+^/eGFP^−^, the cells expressing mCherry reporter only), Q2 (mCherry^+^/eGFP^+^, the cells expressing both mCherry and eGFP), and Q3 (mCherry^−^/eGFP^+^, the cells restoring green fluorescence but lacking mCherry expression). Interestingly, while it was anticipated that all Y66S-corrected cells (eGFP^+^) would also express mCherry reporter, a subset of the corrected cells (Q3) lacked mCherry expression. Although both dsDNA and ssDNA rAAV genomes can be used as HDR templates, productive transduction is essential for HDR as it drives g2 expression. We reasoned this discrepancy to the possible degradation of the dsDNA AAV transduction intermediates, which served as HDR templates prior to reporter expression but lost integrity during the HR processing, resulting in the loss of mCherry expression. Notably, at lower MOI, the proportion of the Q3 cells among the total corrected cells (Q2 + Q3) was higher (Fig. 3C), supporting this hypothesis. Correlation analyses of flow cytometry data revealed a significant positive relationship between the transduction efficiency and the gene editing efficiency (Fig. 3D). Both demonstrated a dose-response effect over the applied vector loads. At the highest MOI of 60K, the total Y66S correction rate reached 13.5 ± 0.8%, with 82.3 ± 5.6% cells being productively transduced. Among these productively transduced cells, the HDR correction rate was calculated to be ∼16.4% (Fig. 3E). Notable, this value represents an average HDR efficiency with a cell pool containing random Y66S *eGFP* integrations. Variations in the efficiency of CRISPR/Cas9 complex access to different genomic loci likely influence the editing outcome in individual cells.

We next pseudopackaged the rAAV2.eGFP630g2-CMVmCh genome into HBoV1 capsid to produce an rAAV2/HBoV1 vector for testing CRISPR-based genome editing in the ALI cultures differentiated from CuFi^Cas9(Y66S)eGFP^ cells. Despite relatively high apical transduction efficiencies as indicated by mCherry^+^ cells, little to no green fluorescence (eGFP restoration) was observed (Fig. 3F). This result was expected because HDR is cell-cycle dependent and most cells, including the basal stem cells, are quiescent in ALI cultures.^59^ Genomic DNA was extracted for PCR analysis to assess NHEJ activity in these cultures. Using a primer set anchored outside the HDR template (Pfw/Prev, Fig. 2A), we obtained a 763-bp amplicon spanning the Y66S codon. The PCR product was cloned into a pCR4blunt-Topo vector, and plasmids from 30 randomly picked colonies were analyzed by Sanger sequencing. Results revealed 11 (36.67%) plasmids contained indels around the CRISPR/Cas9 cut site. Three representative sequences are shown in Figure 3G. Thus, our results confirmed that HDR is inactive in both the quiescent basal cells and well-differentiated epithelial cells in polarized ALI cultures; however, CRISPR-mediated cleavage and the subsequential repair of the DSBs by NHJE are effective.

Collectively, we have established a vector-cell reporter system to evaluate HDR-based gene editing in proliferating airway basal cells, with Y66S corrected indexed by restored green fluorescence. In this system, the CuFi^Cas9(Y66S)eGFP^ reporter cells express Y66S eGFP mutant protein and Cas9, while the rAAV editing vector delivers an sgRNA and an HR template.

### Correcting the F508del CFTR mutation in CuFi^Cas9(Y66S)eGFP^ cells

**Figure 4.**
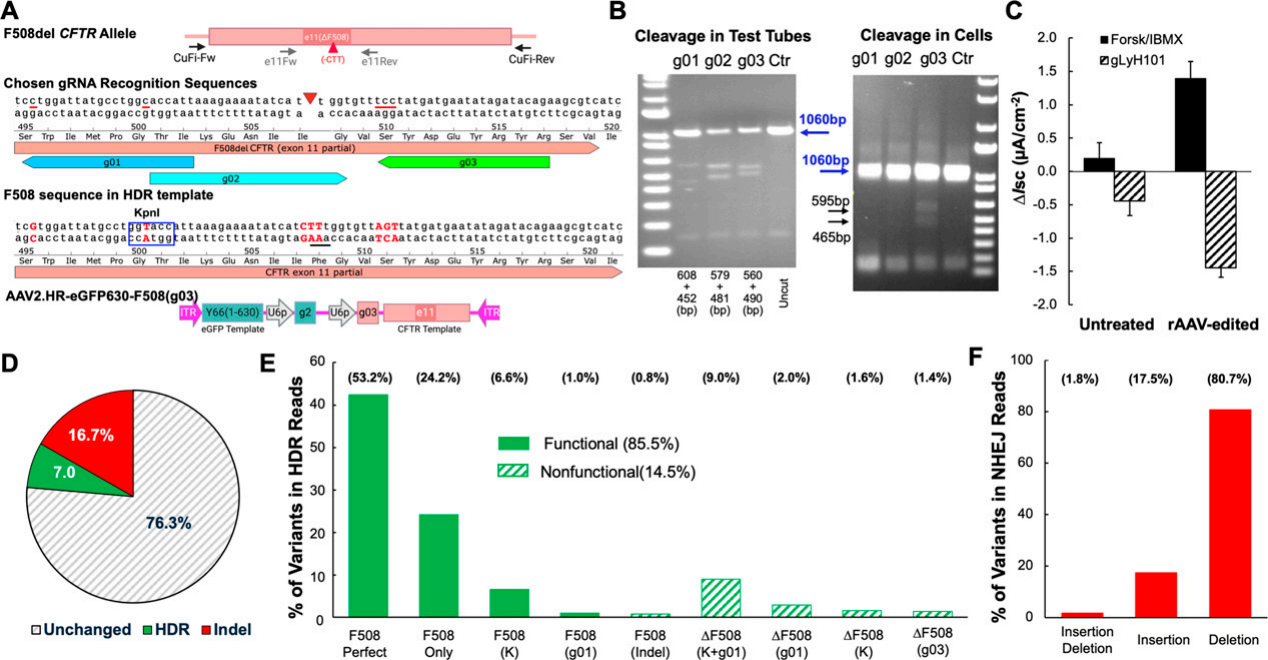
Correction of the F508del *CFTR* mutation in CuFi^Cas9(Y66S)eGFP^ reporter cells. **(A)** Homologous sequences between the F508del allele and HR template within the rAAV vector. Shown are the schematics of the F508del *CFTR* exon with parts of the flanking intronic sequences and the dual-editing rAAV construct (shown is AAV2.HR-eGFP630-F508[g03]) carrying two sets of HR templates and gRNAs for dual-gene editing of *CFTR* and mutant *eGFP* genes, as well as the comparison of the homologous sequences between the F508del allele and HR template within rAAV. *Red triangle* indicated the deletion of the F508 codon. PCR primers for targeted amplicon sequencing are marked with arrows. Primer set CuFi-Fw/Rev specifically amplified products from the genomic DNA, not from the rAAV genome. e11Fw/e11Rev was an internal primer set used to amplify a 310-bp amplicon that was used for NGS of the gene-edited alleles. Three sgRNAs chosen for *in vitro* cleavage are also shown with different color arrow boxes. The Cas9/gRNA complexes recognized the sequences in the F508del allele of CuFi cells but not the HR template, where changes in 8 nucleotides (*red fonts*) reinstalled the F508 codon and mutated the PAMs of g01 and g03, as well as created the *Kpn*I site (*open blue box*) by silent substitutions. The insertion of F508 codon disrupted the PAM of g02. **(B)** Validation of the sgRNA function in rAAV-transduced cells. *Left: in vitro* cleavage assay. A 1.06-kb DNA fragment spanning the target sites was amplified by PCR from CuFi-8 genomic DNA using the CuFi-Fw/CuFi-Rev primer set. The PCR products were incubated with CRISPR/Cas9 ribonucleoprotein (RNP) complex, followed by proteinase K digestion. The reactions were then resolved on a 1% agarose gel. The *blue arrow* points to the dsDNA substrate (uncleaved). The expected band sizes corresponding to cleavage by the RNP complex with each sgRNA are indicated. *Right: in vivo* (cell) assay. Three AAV2/2.5T.HR-eGFP630-F508(g0X) vectors with sgRNA g01, g02, and g03 were produced and used to transduce CuFi^Cas9(Y66S)eGFP^ cells, respectively, at an MOI of 100K. At 2 days post-infection, genomic DNA was extracted from transduced or non-treated cells, and PCR products were amplified using the primer set CuFi-Fw/Rev. Shown is a gel of PCR products following digestion with *Kpn*I; the *blue arrow* points to uncut DNA and the *black arrow* point to the digestion products, indicating genome modification by HDR at the desired site. **(C)** Partial functional correction of CFTR-mediated Cl^–^ transport in polarized epithelial cultures. Two days after transduction of AAV2/2.5T.HR-eGFP630-F508 (g03), cells were seeded onto Transwell® inserts and differentiated at an ALI. Three weeks later, CFTR channel function was assessed in the Ussing chamber. Data represented by the mean ± SD with *N* = 4 of non-transduced cultures and *N* = 7 of transduced cultures. **(D–F)** NGS analysis. A 310-bp amplicon for NGS was produced by PCR with primer set e11Fw/e11Rev, using the 1060-bp PCR product as the template. Amplicon-EZ sequence and analyses were conducted at Azeta Life Sciences. **(D)** Summary of the variant analysis of the 140,681 reads aligned to reference sequences. **(E)** Category and ratio of editing patterns of the 9,853 HDR reads with and without F508 codon insertion. Functional HDR: sequences where the F508 codon was correctly reinstalled and the g03 recognition sequence was mutated. The sequence (partial) of each category is shown in Supplementary Figure S3. (F508 perfect: with all; F508 only: without; F508[K] and F508[g01]: with additional silent nucleotide substitution as indicated.) Non-functional HDR: sequences with the F508 codon reinstalled but alongside indels (F508[indel]) and sequences with indicated silent nucleotide substitutions by HDR but lacking the F508 codon (F508del not changed). **(F)** The mutation types in the reads of mutants with indels generated by NHEJ. NGS, next-generation sequencing.

We engineered a dual-editing AAV vector, rAAV2.HR-eGFP630-F508(g0x) by replacing the mCherry reporter in pAV2.eGFP630g2-CMVmCh with a 995-bp HDR template alongside a U6-driven sgRNA expression cassette for g01, g02, and g03, respectively (Fig. 4A). To validate the selected sgRNAs, we carried out an *in vitro* cleavage assay using a 1.06 kb DNA fragment as a substrate. This fragment was amplified from CuFi-8 genomic DNA using the primer set (CuFi-Fw/CuFi-Rev; Fig. 4A). As shown in Figure 4B (left panel), all three synthesized sgRNAs (g01, g02, and g03) complexed with spCas9 protein successfully cleaved the substrate at the target site, although g01 demonstrated lower efficiency compared with g02 and g03.

The resultant plasmids were used to produce rAAV2.5T vectors for editing both Y66S and F508del mutations. We transduced the CuFi^Cas9(Y66S)eGFP^ reporter cells with each vector at an MOI of 100K. Green fluorescence was observed 24 hours post-transduction, indicating correction of Y66S mutation. No significant differences in Y66S correction efficiency were observed among the three transductions, suggesting all three rAAV vectors were comparably effective in potency (Supplementary Fig. S1). At 2 days post-transduction, cells were seeded onto the Transwell® inserts at a density of 1.5 × 10^5^ cells per insert for differentiation into polarized ALI cultures. Genomic DNA was extracted from ∼1.5 × 10^5^ cells per transduction for PCR analysis. Using the CuFi-Fw/CuFi-Rev primer set, which annealed outside the HDR homology region, we amplified a 1.06-kb DNA product spanning the target site. Sanger sequencing followed by Synthego-Ice (https://ice.editco.bio) analysis revealed indels in 27% of PCR products from cells transduced with rAAV expressing sgRNA g03, but none in cells transduced with the other two vectors. This suggests that rAAV vectors expressing sgRNA g01 and g02 failed to induce cleavage at the target site in the reporter cells.

As the incorporation of *Kpn*I site in the HDR template enables quick diagnostic detection of the HDR events at the F508del *CFTR* locus, the 1.06-kb PCR products from each transduction were digested by *Kpn*I and resolved on an agarose gel. Partial digestion was only observed with the rAAV vector expressing g03 (Fig. 4B, right panel). No evidence of HDR was detected with the other two rAAV vectors expressing sgRNA g01 and g02. Two possible explanations are: (1) the rAAV vectors ineffectively express the sgRNAs g01 and g02 from the U6 promoter, or (2) the Cas9/gRNA complexes did not efficiently access the target sites to induce DSBs. This discrepancy highlights that sgRNA cleavage efficiency in *in vitro* assays does not always translate to performance *in vivo*. Based on these findings, we proceeded to differentiate only the AAV2/2.5T.HR-eGFP630-F508(g03)-transduced cells at an ALI for functional assessment.

Three weeks after air-lift, the fully differentiated ALI cultures from cells that had been previously transduced with AAV2/2.5T.HR-eGFP630-F508(g03) were subjected to Ussing chamber measurements. These cultures exhibited a TEER >1,000 Ω/cm^2^. The acquired *I*sc responses to cAMP agonists and CFTR inhibitor treatments were analyzed and plotted in Figure 4C. The change of CFTR-specific current, Δ*I*sc^GlyH101^, was comparable to ∼26.1% of the level observed in the cultures treated with CFTR modulators (Fig. 4C vs. Fig. 1B). This partial correction of the deficiency in CFTR-specific transepithelial Cl^−^ transport indicated the functional CFTR expression in differentiated epithelial cells where from the F508del allele that had been corrected by HDR. These findings demonstrated that the genetically modified CuFi-8 cells retain their differentiation potential into a pseudostratified airway epithelium and that the subset of F508del-edited cells can also differentiate into functional cell types executing CFTR channel activity.

To assess the editing rate and details of HDR correction at the targe site of the F508del allele, we performed NGS. A 308-bp amplicon was generated by nested PCR using the e11Fw/e11Rev primer set. The template for this second amplification was the 1.06-kb PCR product obtained from the transduced cells using the CuFi-Fw/CuFi-Rev primer set, as described earlier. Among a total of 140,681 reliable reads, 76.3% (107,304 reads) perfectly matched the reference sequence (unchanged), while 7.0% (9,853 reads) were edited by HDR and 16.7% (23,524 reads) contained indels resulted from NHEJ of the CRISPR-induced DSBs at the cut site (Fig. 4D). Further sequence analyses revealed that 85.1% of the HDR-edited sequences were derived from the F508-restored alleles, representing 6.0% of the total reads. Among these sequences, 76 (0.8% of the HDR-edited reads) contained indels at either the sgRNA g03 recognition site or near the F508 codon, disrupting the *CFTR* open reading frame and rendering them non-functional. Other HDR-edited sequences categorized as non-functional included those that incorporated the silent base substitutions from the template but lacked the CTT insertion required to restore the F508 codon. Of the HDR sequences considered functional for WT CFTR expression, most (52.2% of the HDR reads) were perfectly edited according to the HDR template, while other subsets lacked the silent base substitutions introduced the HDR template at the g01 recognition sequence or the *Kpn*I site or both, in varying proportions (Fig. 4E). In our previous study on editing the G551D mutation at the ferret *CFTR* locus in primary airway basal cells using rAAV, we observed a significantly lower rate of HDR compared with NHEJ.^27^ Similarly, in this study, the rAAV-mediated correction rate of the F508del mutation via HDR in the CuFi-8 derivatives was also lower than that of NHEJ. Interestingly, over four-fifths of the NHEJ-incurred sequences in this study involved deletions (Fig. 4F), contrasting with our previous observation that three-fourths of NHEJ mutations in edited ferret airway basal cells were associated with insertions. Both studies used rAAV to deliver the sgRNA and HDR template to airway basal cells integrated with Cas9 expression in a cell pool. It is unclear whether the observed differences in NHEJ mutagenesis patterns are due to species-specific variations or locus-specific effects, but the latter appears more likely.

### Dual-gene editing of F508del CFTR and Y66S eGFP mutations in proliferating the CuFi-reporter cells

In our previous study on correcting the G551D mutation in the ferret *CFTR* locus, we also used a lentiviral vector to integrate stable expression of Cas9 and Y66SeGFP into the airway basal cells isolated from the *CFTR*^G551D^ CF ferret.^27^ We observed a high frequency of simultaneous gene editing at both the G551D mutation in *CFTR* and the Y66S mutation in *eGFP*. However, NGS analyses of HDR- and NHEJ-mediated editing, as well as assessments of CFTR functional restoration, were focused solely on eGFP^+^ cells. In this study, we expanded the analyses to include both eGFP^+^ and eGFP^−^ populations.

**Figure 5.**
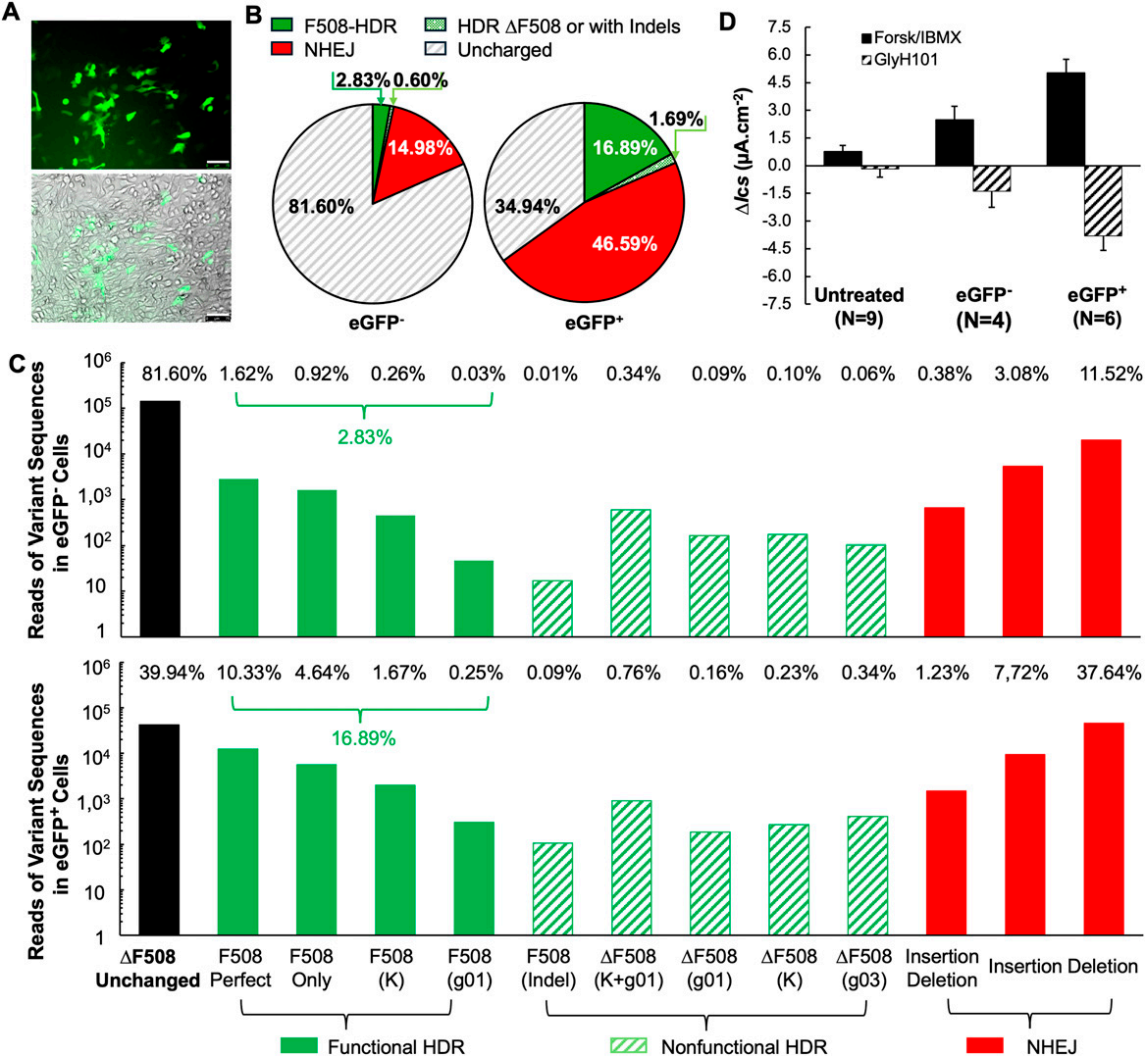
Dual-correction of the F508del *CFTR* and Y66S *eGFP* in CuFi^Cas9(Y66S)eGFP^ reporter cells. **(A)** Fluorescence of eGFP was visualized in gene-edited cells. CuFi^Cas9(Y66S)eGFP^ cells were transduced with AAV2/2.5T.HR-eGFP630-F508(g03) at an MOI of 100K. At 2 days post-infection, the cells were passaged onto 100-mm dish for expansion followed by FACS to separate the eGFP^+^ and eGFP^–^ populations. Representative images captured 7 days before cell sorting are shown. Scale bar: 75 µm. **(B–C)** Amplicon-EZ sequencing analysis of the F508del *CFTR* locus in eGFP^+^ and eGFP^–^ cells. **(B)** Summary of 120,491 reads of the eGFP^+^ cells and 175,043 reads of the eGFP^–^ cells aligned to the reference sequence. The major editing patterns and their respective proportions are shown **(C)** Variant analyses of the mutation types identified in the NGS data from eGFP^–^ (*upper panel*) and eGFP^+^ cells (*lower panel*). The percentage of reads in each category is indicated. Functional HDR: sequences where the F508 codon was correctly reinstalled, with or without additional silent nucleotide substitution; Non-functional HDR: sequences with the F508 codon reinstalled alongside indels and F508del sequences with indicated silent nucleotide substitutions by HDR. The HDR-edited sequence of each category is shown in Supplementary Figure S3. NHEJ: mutants with insertion or deletion or both, generated through NHEJ. **(D)** Partial functional correction of CFTR-mediated Cl^–^ transport in polarized epithelial cultures derived from the eGFP^+^ and eGFP^–^ cell populations from the rAAV-transduced cells. CFTR-mediated Cl^–^ transport was quantified as the Δ*I*sc following activation by Forsk/IBMX and inhibition by GlyH101. Data are represented by the means ± SD (*N* is indicated for each group). FACS, fluorescence-activated cell sorting.

Two days after transduction with AAV2/2.5T.HR-eGFP630-F508(g03), CuFi^Cas9(Y66S)eGFP^ cells on one well of a 6-well plate were transferred to a 100-mm dish for expansion. Over the subsequent 5 days, the cells reached subconfluence, during which colony expansion of the edited cells was visually observed as clusters of eGFP^+^ cells (Fig. 5A). At 7 days post-transduction, eGFP^+^ and eGFP^−^ cells were isolated using FACS. Of the total viable cells, 320,929 cells (12.5%) were eGFP^+^. These cells, along with 956,322 eGFP^−^ cells (33.5%), were collected for assessments of editing efficiencies at the *CFTR* locus using NGS and for restoration of CFTR function in Ussing chamber measurement. To ensure eGFP^+^ and eGFP^−^ cells were well-separated, approximately 54.0% of cells lying between the two populations were excluded from further experiments (Supplementary Fig. S2).

After FACS isolation, the cells were expanded for an additional week and then seeded onto Transwell® inserts for differentiation at an ALI. At this time point, genomic DNA was extracted from subsets of eGFP^−^ and eGFP^+^ cells (1.5 × 10^5^ each) for nested PCR to generate a 308-bp amplicon for NGS analysis, as described above. Consistent with our previous observation, NGS results reveal a high frequency co-editing at the two loci in the eGFP^+^ cells. Specifically, 65.06% of sequences were edited via HDR or by NHEJ. Among these, 25.97% of sequences (16.89% of total reads) contained the F508 codon insertion without disrupting the ORF, representing the corrected *CFTR* allele with potential functional restoration. In contrast, the eGFP^−^ population exhibited a lower editing frequency of 18.40% at the *CFTR* locus, with 3.42% and 14.98% of sequences subjected to HDR- and NHEJ-editing, respectively. The HDR-edited sequences containing the F508 codon insertion with potential functionality (no indels) accounted for only 2.8% of the total reads in this population (Fig. 5B). Notably, in both eGFP^+^ and eGFP^−^ populations, the predominant sequences containing the F508 codon repaired precisely according to the HR template: 47.35% of the HDR-edited sequences in eGFP^−^ cells and 55.99% in eGFP^+^ cells, closely matching the 52.46% observed in the unsorted cell population at 2 days post-transduction (Fig. 4E). Subsets of HDR-edited sequences that lacked template-derived nucleotide substitutions at either the gRNA recognition site, the *Kpn*I site, or both were also identified. These imperfect HDR-edited sequences, along with NHEJ-edited sequences, exhibited similar distribution patterns across both sorted populations (Fig. 5C), mirroring those observed in the unsorted cell population at 2 days post-transduction (Fig. 4E, F).

Next, we evaluated CFTR-specific transepithelial Cl^−^ transport in the well-differentiated ALI cultures derived from eGFP^+^ and eGFP^−^ cells using Ussing chamber measurements. In ALI cultures derived from eGFP^−^ population, CFTR channel activity (Δ*I*sc^GlyH101^) reached 25.2% of the level achieved with CFTR modulator treatment, despite a low genomic correction rate of only 2.8% for F508 codon restoration. Notably, in cultures derived from eGFP^+^ population, Δ*I*sc^GlyH101^ reached 68.7% of the level achieved with the CFTR modulator treatment (Fig. 5D vs. Fig. 1B). This finding suggests a non-linear correlation between CFTR functional restoration and genomic correction rates in basal cells transduced with the rAAV editing vector. While cultures from eGFP^+^ cells exhibited a sixfold higher correction rate of 16.89% compared with eGFP^−^ cells (2.8%), their CFTR channel activity was only 2.7-fold higher.

## DISCUSSION

While FDA-approved CFTR modulator therapies can rescue the dysfunctional CFTR protein, patients with CF who produce minimal to no CFTR or who cannot tolerate modulators still rely on symptomatic therapies.^60,61^ For eligible patients, lifelong commitment of pharmaceutical management (*e.g.,* Trikafta® at ∼$322,000 annually per patient) imposes a substantial financial burden on families and health care systems. Gene therapies for CF lung disease, regardless of a patient’s genotype, offer a promising path toward an ultimate cure. However, CF gene therapy faces unique challenges. Unlike other genetic diseases with FDA-approved gene addition therapies,^62–66^ CF is a complex disease and CFTR expression is tightly regulated in a cell-type-specific manner within the respiratory system coordinating ASL hydration, mucociliary clearance, and innate immunity in airways.^53,67–69^ Restoring normal airway homeostasis in CF lungs via *CFTR* addition may require regulated expression across multiple cell types in both conducting airways and pulmonary parenchyma. Furthermore, most surface epithelial cells accessible to *CFTR* transfer vectors are terminally differentiated with finite lifespans, sustained CFTR expression would likely necessitate periodic repeat dosing throughout a patient’s life.^10,70^

Gene editing holds the potential for permanent correction of defective genes without altering their natural expression patterns, positioning it as an innovative solution for CF gene therapy beyond traditional *CFTR* replacement. *CFTR* gene editing in terminally differentiated epithelial cells restores CFTR expression for each cell’s lifespan, thus, like the treatment of *CFTR* addition, repeat dosing is required. However, permanent correction is achievable by targeting lung progenitor cells capable of self-renewal and differentiation into various CFTR-expressing cell types. Autologous cell-based therapy could also become viable, providing challenges such as maintaining the multipotency of edited basal cells during *ex vivo* expansion, achieving reliable engraftment, and ensuring long-term persistence in the recipient airways can be resolved.^71,72^

Our previous study demonstrated that AAV-mediated HDR editing in the proliferating basal cells isolated from *CFTR*^G551D^ ferret enabled these AAV-transduced cells to retain multipotency and differentiate into pseudostratified airway epithelium when cultured at an ALI. Notably, *CFTR* correction in a small population (3.08%) achieved greater functional recovery exceeding the observed gene editing frequency, restoring ∼26.0% of the Δ*Isc*^GlyH101^ seen in non-CF cultures.^27^ In this study, ALI cultured derived from the rAAV-transduced CuFi^Cas9(Y66S)eGFP^ cells with a small fraction of cells (5.9%) carrying corrected F508 *CFTR* allele, demonstrated a level of 26.1% functional restoration relative to CFTR modulator treatment. Following limited expansion, these rAAV-transduced cells were sorted into two populations based on Y66S *eGFP* reporter correction. ALI cultures derived from eGFP^−^ cells, which had 2.8% F508 *CFTR* copies at genomic level, exhibited a level of functional recovery equivalent to ∼25.2% of modulator treatment efficacy. In contrast, eGFP^+^ cultures, displaying a sixfold enrichment in corrected F508 *CFTR* copies (16.89%), showed only a 2.7-fold increase in functional restoration. This discrepancy suggests that factors beyond genomic correction rates may influence the efficiency of functional restoration. Since gene correction was performed in differentiation-competent reporter cells, post-editing cell dynamics could influence functional outcomes, *i.e.*, CFTR channel activity, in the ALI cultures. These include the differential proliferation or selective advantage of corrected versus non-corrected cells during their division and differentiation at an ALI, as well as the off-target effects on the differentiation potential of the F508del-corrected cells. While the incorporation of an eGFP reporter aids in enriching the F508del *CFTR*-edited cells via FACS, dual-editing at two individual loci might inadvertently impact the cell differentiation potential of these cells. Moreover, targeting efficiency is influenced by the delivery vector, the type of targeted cell, and the target loci. For instance, we observed a correction rate for Y66S *eGFP* that was approximately twofold higher than that of F508del *CFTR*.

HDR-based CFTR corrections have demonstrated the capacity to address a wide range of mutations, including correcting missense or nonsense mutations in exons, ablating splice mutations, and inserting a CFTR expression cassette into a genome safe harbor using a DNA donor template provided alongside CRISPR components.^73–75^ Achieving efficient gene editing in targeted cells is crucial for the success of these methods. However, our NGS analyses revealed that NHEJ-mediated mutagenesis at the target site was more prevalent than HDR-based correction, indicating that NHEJ predominates over HDR in repairing the CRISPR-induced DSBs in airway basal cells, even in the presence of HR templates. This high rate of NHEJ mutations occurred at target site and NHEJ preference largely restricts HDR-based correction efficiency. Pharmaceutical intervention to shift cellular DNA repair pathways to favor HDR over NHEJ could potentially improve editing outcome.^31,76–78^ The CuFi^Cas9(Y66S)eGFP^ reporter system enables rapid assessment of HDR efficiency at the Y66S *eGFP* locus through the restoration of green fluorescence. By eliminating the variables associated with co-delivery of a Cas9-expressing rAAV vector, the reporter system developed in this study offers a streamlined platform for approach validation and optimization. Additionally, it facilitates drug screening to identify small molecules that can modulate DNA repair pathways to enhance gene editing efficiency.

Despite its potential, the *in vivo* application of HDR faces challenges due to its cell-cycle-dependency, which renders it ineffective in well-differentiated airway epithelium. In contrast, the NHEJ-based HITI is suitable for editing a broad range of cell types, including both diving and non-dividing cells, and can be applied both *in vivo* and *ex vivo*. For example, HITI has been used to insert a *CFTR* mega exon into the intronic sequence via promoter trap, bypassing mutations located downstream the edited site.^29^ This strategy represents a potential universal therapeutic strategy for most patients with CF, regardless of their genotypes. Our study confirmed that NHEJ is effective in HAE-ALI cultures. While the lentiviral vector and rAAV editing vectors described in this study can be adapted to create similar reporter cell lines for developing and optimizing editing strategies or drug screening to improve editing efficiency, our current reporter system is strictly limited to *in vitro* studies focused on HDR. In addition to enhancing editing efficiency, *in vivo* gene editing presents additional challenges compared with *ex vivo* applications, such as the efficient delivery of editing tools to target cells, the risk of off-target mutations, and the immune responses to the editing components. Achieving permanent correction requires targeting progenitor stem cells, which poses particular challenges for therapeutic gene editing in CF. In this context, airway basal cells, which reside beneath the airway surface and typically maintain quiescence under homeostatic conditions, represent a difficult yet essential target. Moving forward, the development of a versatile reporter system in transgenic animal models, capable of *in vivo* evaluating both HDR and HITI outcomes, as well as advanced editing tools such as base editors and prime editors, will be essential for advancing therapeutic genome editing for CF and other genetic disorders.
