# Supplementary material for: Recombinant Adeno-Associated Virus Vector Mediated Gene Editing in Proliferating and Polarized Cultures of Human Airway Epithelial Cells
Source: Hum Gene Ther. 2025 Aug 4;36(15-16):1067–82. doi: 10.1089/hum.2024.260 (PMC12409266; doi:10.1089/hum.2024.260)
Supplement: Supplementary Figure S1 [file hum.2024.260_supplementary_figures1.pdf]

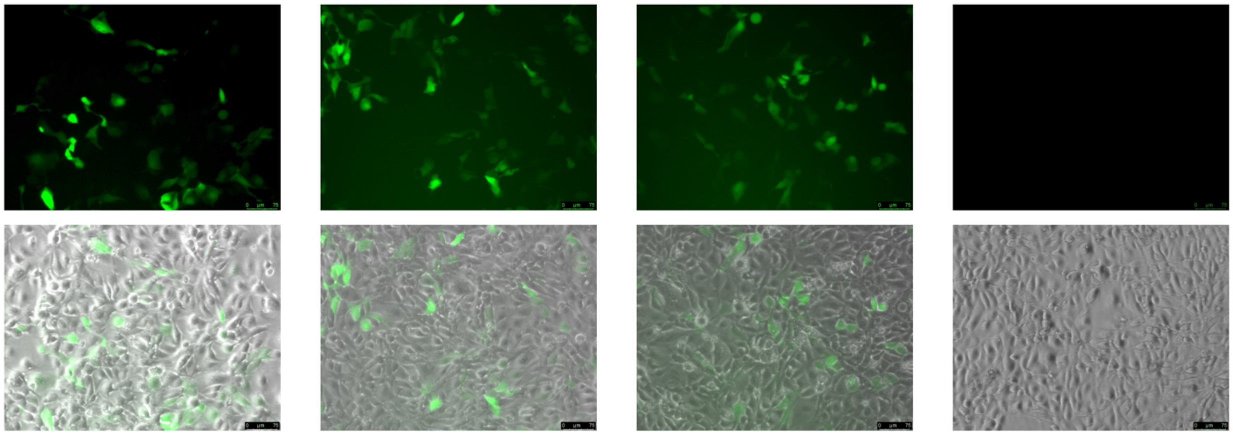

Supplementary Figure 1. rAAV dual-editing vectors.
rAAV2.HR-eGFP630-F508(g01), rAAV2.HR-eGFP630-F508(g02) and rAAV2.HR-eGFP630-F508(g03) share the same genome structure but differ in their sgRNAs (g01, g02 and g03), which target three different sites around or near the F508del mutation at the *CFTR* locus. $\mathrm{CuFi}^{\mathrm{Cas9(Y66S)eGFP}}$ reporter cells were transduced with the AAV2.5T capsid-pseudopackaged vectors at an MOI of 100K, respectively. Images were captured two days post-infection (scale bar: 75μm).
