# Supplementary material for: Recombinant Adeno-Associated Virus Vector Mediated Gene Editing in Proliferating and Polarized Cultures of Human Airway Epithelial Cells
Source: Hum Gene Ther. 2025 Aug 4;36(15-16):1067–82. doi: 10.1089/hum.2024.260 (PMC12409266; doi:10.1089/hum.2024.260)
Supplement: Supplementary Figure S2 [file hum.2024.260_supplementary_figures2.pdf]

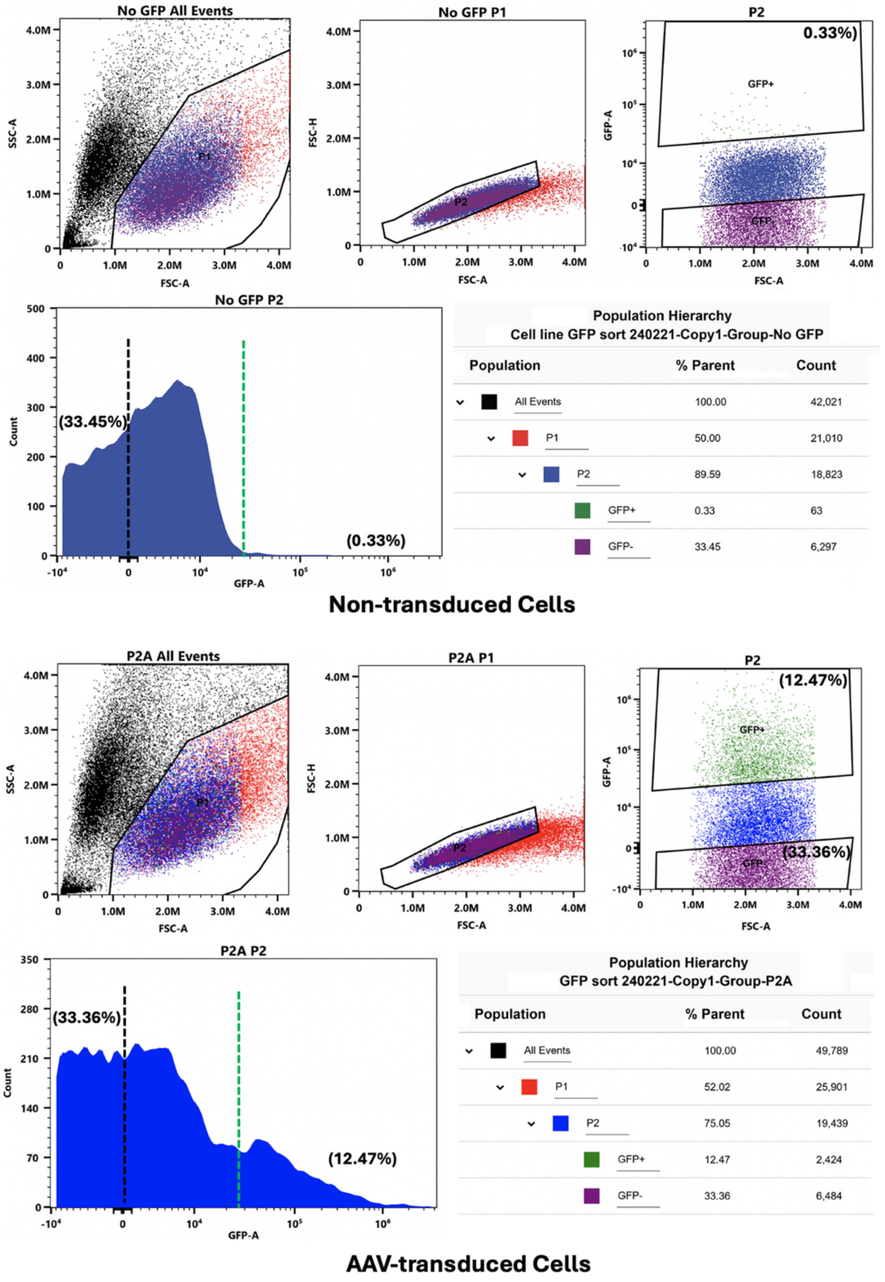

Supplementary Figure 2. Gating strategy for sorting eGFP^{+} cells by flow cytometry.
eGFP^{+} and eGFP^{-} populations from the rAAV-transduced CuFi$^{Cas9(Y66S)eGFP}$ cells were sorted using fluorescence-activated cell sorting (FACS) on the Cytek Aurora^{TM} CS system. Non-transduced CuFi$^{Cas9(Y66S)eGFP}$ cells were used as control. Cells of interest were gated based on forward scatter (FCS) and side scatter (SSC), and single cells were further gated based on forward scatter height (FCS-H) and forward scatter area (SSC-A). eGFP-expressing cells in the P2 populations were identified as being outside the fluorescence range of the non-transduced cells, as detected with the 488 nm laser (with intensity above the green dashed line). To ensure a clear separation of the eGFP^{+} and eGFP^{-} cells, cells located between the two populations (to the right of the black dashed line and to the left of the green dashed line) were excluded from further experiments.
