# Supplementary material for: Recombinant Adeno-Associated Virus Vector Mediated Gene Editing in Proliferating and Polarized Cultures of Human Airway Epithelial Cells
Source: Hum Gene Ther. 2025 Aug 4;36(15-16):1067–82. doi: 10.1089/hum.2024.260 (PMC12409266; doi:10.1089/hum.2024.260)
Supplement: Supplementary Figure S3 [file hum.2024.260_supplementary_figures3.pdf]

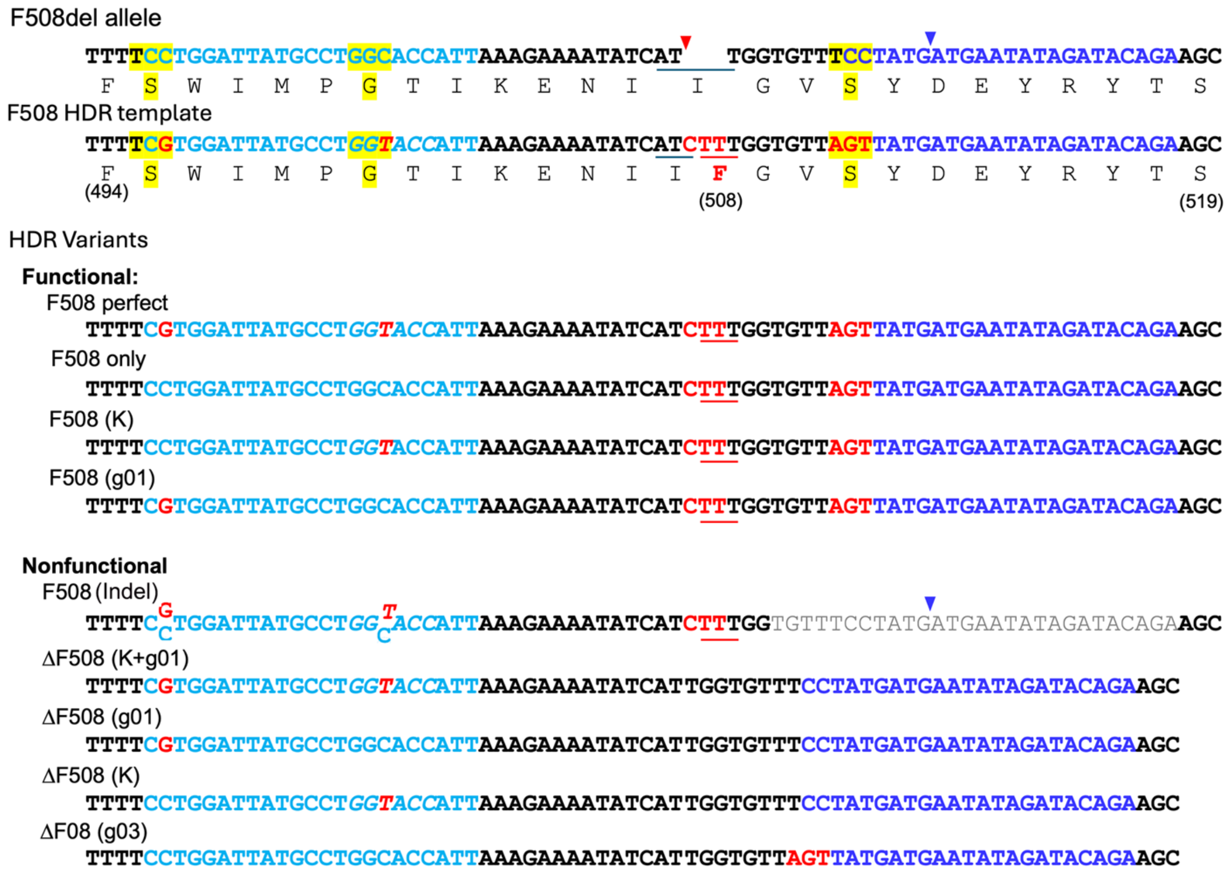

Supplementary Figure 3. Categories of variants resulting from homology-directed repair (HDR). In the F508 HDR template, red fonts indicate the three-nucleotide insertion (CTT) used to reinstall the F508 codon (TTT, underlined in red) and silent mutations designed to disrupt the PAM sequences of sgRNAs g01 and g03 or introduce the *Kpn*I restriction site (italicized). Cyan fonts represent the recognition sequence of sgRNA g01, while blue fonts represent the recognition sequence of sgRNA g03. The red triangle points to the insertion site, and the blue triangle marks the CRISPR cut site within g03. Codons with silent mutations in the template and the corresponding codons in the F508del allele are highlighted in yellow. HDR-edited sequences are categorized, with the substituted nucleotides marked in red, and their proportions in the NGS reads from two different experiments are presented in **Figure 4E** and **Figure 5C**. Sequences in four categories containing F508 codon are expected to express functional CFTR protein. Among the five kinds of nonfunctional HDR sequences, the F508 (Indel) category represents the sequences where the F508 codon is present but various indels have occurred at the CRISPR cut site (grey fonts indicate highly frequent insertion or deletion there, not actual sequences), while the other four lack the F508 codon.
